# Allometric trajectories of body and head morphology in three sympatric Arctic charr (*Salvelinus alpinus* (L.)) morphs

**DOI:** 10.1002/ece3.3224

**Published:** 2017-08-08

**Authors:** Marianne Knutsdotter Simonsen, Anna Siwertsson, Colin Ean Adams, Per‐Arne Amundsen, Kim Præbel, Rune Knudsen

**Affiliations:** ^1^ Department of Arctic and Marine Biology Faculty of Biosciences, Fisheries and Economics UiT The Arctic University of Norway Tromsø Norway; ^2^ Scottish Centre for Ecology and the Natural Environment IBAHCM, University of Glasgow Glasgow UK; ^3^ Faculty of Biosciences, Fisheries and Economics The Norwegian College of Fishery Science UiT The Arctic University of Norway Tromsø Norway

**Keywords:** niche segregation, phenotypic diversity, polymorphism, salmonids

## Abstract

A study of body and head development in three sympatric reproductively isolated Arctic charr (*Salvelinus alpinus* (L.)) morphs from a subarctic lake (Skogsfjordvatn, northern Norway) revealed allometric trajectories that resulted in morphological differences. The three morphs were ecologically assigned to a littoral omnivore, a profundal benthivore and a profundal piscivore, and this was confirmed by genetic analyses (microsatellites). Principal component analysis was used to identify the variables responsible for most of the morphological variation of the body and head shape. The littoral omnivore and the profundal piscivore morph had convergent allometric trajectories for the most important head shape variables, developing bigger mouths and relatively smaller eyes with increasing head size. The two profundal morphs shared common trajectories for the variables explaining most of the body and head shape variation, namely head size relative to body size, placement of the dorsal and pelvic fins, eye size and mouth size. In contrast, the littoral omnivore and the profundal benthivore morphs were not on common allometric trajectories for any of the examined variables. The findings suggest that different selective pressures could have been working on traits related to their trophic niche such as habitat and diet utilization of the three morphs, with the two profundal morphs experiencing almost identical environmental conditions.

## 1 INTRODUCTION

A major goal of evolutionary ecology is to understand how and why organisms diversify (Adams & Nistri, 2010; Pfennig et al., 2010). Diversification and speciation often follow colonization of new environments (Orr & Smith, 1998; Price, Qvarnström, & Irwin, 2003) and an intermediate step toward speciation is often the evolution of morphs that utilize different resources, such as habitat and food (Pfennig et al., 2010; Smith & Skúlason, 1996). Thus, resource polymorphism is a recognized step in ecological speciation, especially when it occurs in sympatry (Berlocher & Feder, 2002; Schliewen, Tautz, & Pääbo, 1994; Via, 2001) and may eventually lead to reproductive isolated populations of eco‐morphs (Præbel et al., 2013; Rundle & Nosil, 2005; Wimberger, 1994). Factors that may promote resource polymorphism are vacant niches, habitat variability, and relaxation of interspecific competition (Smith & Skúlason, 1996). Different mating strategies among males can also lead to polymorphism, for example, in North American sunfishes (*Lepomis*: Centrarchidae; Gross, 1982) and the dynastine beetle (*Podischnus agenor*; Eberhard, 1982). Polymorphism is usually seen in species‐poor communities, and it is probably linked to niche expansion in the absence of interspecific competitors and predators (Robinson & Wilson, 1994). Many species‐poor communities with polymorphic fish species are found in the northern hemisphere, particularly in postglacial lakes. Examples include polymorphic brown trout (*Salmo trutta* L.) in Lake Bunnersjöarna in Sweden (Ryman, Allendorf, & Ståhl, 1979), rainbow smelt (*Osmerus mordax*) in Lochaber Lake (Nova Scotia), Lake Utopia (New Brunswick) and Onawa Lake (Maine; Taylor & Bentzen, 1993), European and lake whitefish (*Coregonus* spp.) in northern Fennoscandia and Canada (Bernatchez, Vuorinen, Bodaly, & Dodson, 1996; Siwertsson et al., 2010) and three‐spined stickleback (*Gasterosteus aculeatus* L.) in British Columbia (McPhail, 1992). Thus, postglacial lakes represent important ecosystems where mechanisms of diversification and speciation can be studied (Skulason, Snorrason, & Jonsson, 1999).

The morphology of an individual influences that individuals' ability to perform key tasks in its daily life (Wainwright, 1991) and large differences in relative fitness may be caused by small morphological differences (Parsons, Sheets, Skúlason, & Ferguson, 2011). A common method used to detect and quantify morphological differences is to use geometric morphometrics to explore shape (Adams, Rohlf, & Slice, 2004, 2013). This technique allows exploration of evolutionary and developmental questions by comparison of populations or by studying ontogenetic changes (Parsons, Robinson, & Hrbek, 2003). Shape changes occurring during an individual`s lifetime forms the basis for the potential for adaptive evolution (Klingenberg & Spence, 1993). Organisms can change shape as they develop by changes in the relative growth rate of morphological features (Urošević, Ljubisavljević, & Ivanović, 2013) and/or alterations in the timing of developmental events (Eiríksson, Skulason, & Snorrason, 1999). One approach to describing morphological changes in shape as an individual grow is to study developmental trajectories (Klingenberg, 1998; Sheets & Zelditch, 2013; Webster & Zelditch, 2005). Growth trajectories manifested as allometric scaling are important in evolutionary processes as differences in these trajectories have the potential to provide for the expression of different phenotypes which increase the potential for evolution in new directions (Frankino, Zwaan, Stern, & Brakefield, 2005; Klingenberg, 1998).

**Figure 1:**
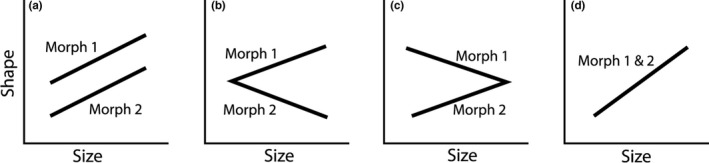
Possible patterns of allometric trajectory comparisons between two morphs using PC scores as measures of size: (a) phenotypic parallelism; (b) phenotypic divergence; (c) phenotypic convergence, and (d) common allometric trajectory. For further explanations, see the text

Allometric trajectories can potentially change direction, shift sideways through lateral transposition or they can be extended or truncated (Klingenberg, 1998). If the trajectories change direction, then a dissociation of the feature measured and age or size during the period being studied has occurred. A lateral transposition in contrast suggests that the dissociation likely has occurred earlier in development when there often is higher evolutionary flexibility for changes in shape (Klingenberg, 1998). In these circumstances, the morphology of one group will resemble shorter or longer individuals of another group (Sheets & Zelditch, 2013). Conserved trajectories indicate that ancestral growth trajectories are maintained (Klingenberg, 1998). Based on these, there are at least three possible allometric pathways that could result in the formation of alternative morphological groups (Figure 1a–c), and a fourth where the morphologies of the studied groups are on the same trajectory (Figure 1d). Phenotypic parallelism occurs where two morphs are on the same allometric shape trajectory, but the trajectories have different starting points; thus, there is a lateral transposition (Figure 1a). Phenotypic divergence over time occurs when two morphs are on different allometric trajectories that diverge with the size of the individuals (Figure 1b). Phenotypic convergence occurs when two morphs are on different allometric trajectories, where the trajectories have different starting points but converge with increasing size (Figure 1c). Finally, with common allometric trajectories, two morphs are on the same allometric shape trajectory (overlapping; Figure 1d). In this case, the two groups being studied can have the same size range, or the trajectories can be extended or truncated (Klingenberg, 1998). Ultimate differences in expressed shape are the result of different allometric trajectories or different shape starting points, whereas converging trajectories will reduce any initial differences.

Closely related species or morphs are often morphologically similar early in ontogeny and may diverge later in ontogeny to produce distinct adult morphologies (Richardson, 1999). This is often seen for primates (Mitteroecker, Gunz, Bernhard, Schaefer, & Bookstein, 2004; Richtsmeier, Corner, Grausz, Cheverud, & Danahey, 1993; Schultz, 1924) and for fish such as Eurasian perch (*Perca fluviatilis*; Svanbäck & Eklöv, 2002). However, species or morphs may show a converging morphology if they experience more similar environments at later life stages, for instance seen for three‐spined sticklebacks and European cave salamanders (Family: Plethodontidae; Adams & Nistri, 2010; Oke et al., 2016).

**Figure 2:**
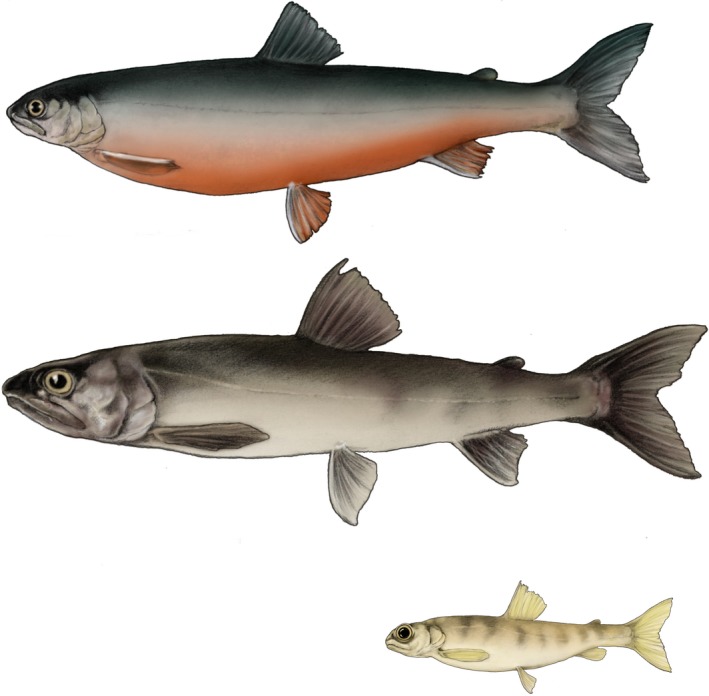
Drawing of the three morphs in Lake Skogsfjordvatn, showing their typical appearance. Uppermost individual LO‐morph, middle PP‐morph, and lower PB‐morph. Drawing: Sigrid Skoglund

Fish may respond more readily to environmental complexity than other vertebrates because of their flexibility in life history, growth rate, and body size (Snorrason et al., 1994). In this study, a morphologically diverse fish species, Arctic charr (*Salvelinus alpinus* (L.)), is studied in order to explore allometric patterns of shape change among sympatric morphs. The study lake, Skogsfjordvatn, northern Norway, supports three reproductively isolated morphs that differ in habitat use, diet, and life history traits (Figure 2; Knudsen et al., 2016; Siwertsson, Refsnes, Frainer, Amundsen, & Knudsen, 2016; Skoglund, Siwertsson, Amundsen, & Knudsen, 2015; Smalås, Amundsen, & Knudsen, 2013). The morphs are referred to as the littoral spawning omnivore morph (LO), the profundal spawning benthivore morph (PB), and the profundal spawning piscivore morph (PP). The PB morph occurs exclusively in deep water, the PP morph appears to spend most time in the profundal zone, and the LO morph typically occurs in the littoral and pelagic zones. The PB morph is slow growing and attains a maximum size of 12–15 cm, whereas the other two morphs grow larger (Smalås et al., 2013). Throughout its life span, the PB‐morph has clear, dark finger marks along its body sides, while the other two morphs only exhibit these marks at the earliest life stages. Morphologically the body of the PB‐morph appears to be deeper than the bodies of the LO‐ and PP‐morphs at the same fork lengths. Focusing at head structures, the PP‐ and PB‐morphs seemingly have relatively larger eyes and mouths than the LO‐morph. Also the breeding coloration of the morphs differ, with the LO‐morph showing typically strong, red colors on their bellies, while the PP‐ and PB‐morphs do not get clear signs of breeding colors. The diet of the PB‐morph is dominated by profundal benthos, while the PP‐morph initially feeds on profundal benthos but becomes piscivorous at a fork length around 20 cm. Zooplankton is the main prey of the LO‐morph under 25 cm in fork length (Knudsen et al., 2016), but fish become more important in the diet with increasing length of the morph.

We examined the developmental pathways leading to the expression of different morphologies in the three reproductively isolated morphs by comparing the development of body and head shape. A basis of the study is that the three morphs are genetically different from each other. We predict that: (1) The PP‐ and PB‐morphs are on common or slightly divergent allometric trajectories because they utilize similar habitats and diets at similar sizes, (2) The LO‐ and PP‐morphs are on convergent trajectories because they have diets that become more dominated by piscivory during growth, although they utilize different principal habitats, and (3) The LO‐ and PB‐morphs do not share common allometric trajectories because they utilize different habitats and diets.

## 2 MATERIALS AND METHODS

### 2.1 Study area

Skogsfjordvatn (69°56ʹ24ʺN, 19°10ʹ00ʺE) is a 13.6 km² oligotrophic and dimictic lake at 17 m above sea level on Ringvassøya, northern Norway. The lake is normally ice‐covered from December to May. The maximum depth is 120 m, and most of the lake has depths between 30 and 65 m with well‐differentiated littoral, pelagic, and profundal zones. The lake is connected to the sea via a 1‐km‐long outlet river. The fish community consists of Arctic charr, brown trout, three‐spined stickleback, Atlantic salmon, and European eel (*Anguilla anguilla* (L.); Smalås et al., 2013).

### 2.2 Fish sampling

Sampling took place monthly from August 2011 to January 2012 (*n* = 200) and in September and October 2012 (*n* = 49) using monofilament multi‐mesh gillnets with mesh sizes that varied from 5 to 55 mm knot to knot. In the littoral (0–12 m depth) and profundal (>25 m depth) zones, 1.5‐m‐deep bottom nets were used, whereas 6‐m‐deep floating nets were employed in the pelagic zone above >30 m water depths. Fish were initially classified to morph in the field by examining head and body shape and color and a sample of fin tissue were obtained for subsequent genetic classification. In the laboratory, fork length (FL) of each fish was measured to the nearest 0.1 cm.

### 2.3 Phenotypic and genetic morph classification

All individuals were assigned to one morph category based on visual morphological traits according to Skoglund et al., 2015 (also see Figure 2). This included the overall coloration, the general head and body shape, the relative eye size, the mouth position and size, and the curvature from the head to the snout.

Genetic classification of all individuals was performed via genetic assignment using a panel of seven validated microsatellites (see Appendix S2 for details). This panel of microsatellites is suited for discriminating the three charr morphs, but is not informative for the inference of adaptive traits and the genetics of allometric processes due to the neutrality of the loci. To confirm the existence of three charr morphs in the dataset, individuals classified as adults and with the phenotypic characteristic of the three charr morphs were analyzed using Bayesian clustering as implemented in STRUCTURE 2.3.4 (Hubisz, Falush, Stephens, & Pritchard, 2009; Pritchard, Stephens, & Donnelly, 2000). We used a model that assumed admixture and correlated allele frequencies between *K* clusters and burn‐ins of 30,000 and MCMC replications of 50,000 at values of *K *=* *1–6. No prior information was provided to the model, and the model was run 10 times at each *K* to confirm consistency of log‐likelihood probabilities. The most likely number of morphs was evaluated as the highest ln Pr(*Χ*|*Κ*) and Δ*K* using STRUCTURE HARVESTER (Earl, 2012). Adult individuals with membership coefficients, *q*, lower/higher than 0.1/0.9 were used to establish three reference populations (LO, *n* = 45; PB, *n* = 42; PP, *n* = 45). These reference populations were then used to validate the phenotypical classification of the remaining ontogenetic stages (*n* = 143). The genetic assignment was performed with GeneClass2 (Piry et al., 2004), using Bayesian computation (Rannala & Mountain, 1997). Monte–Carlo resampling (Paetkau, Slade, Burden, & Estoup, 2004) using 10,000 simulated individuals and α = .01 was also employed to obtain probabilities of the assignments. The assignment was confirmed by an additional STRUCTURE analysis, using similar settings as above, where all (*N* = 275) individuals were included. As some individuals showed signatures of some admixture, conservative *q* value thresholds of 0.3/0.7 (Vähä, Erkinaro, Niemelä, & Primmer, 2007; Vähä & Primmer, 2006; Warnock, Rasmussen, & Taylor, 2010) were used for evaluation for the membership of each individual to each of the *K* clusters. The assignment of each individual obtained by GeneClass2 and STRUCTURE was subsequently manually compared to ensure consistency, and individuals were omitted from the subsequent analyses if the assignment was not consistent in all three approaches.

### 2.4 Morphological analyses

**Figure 3:**
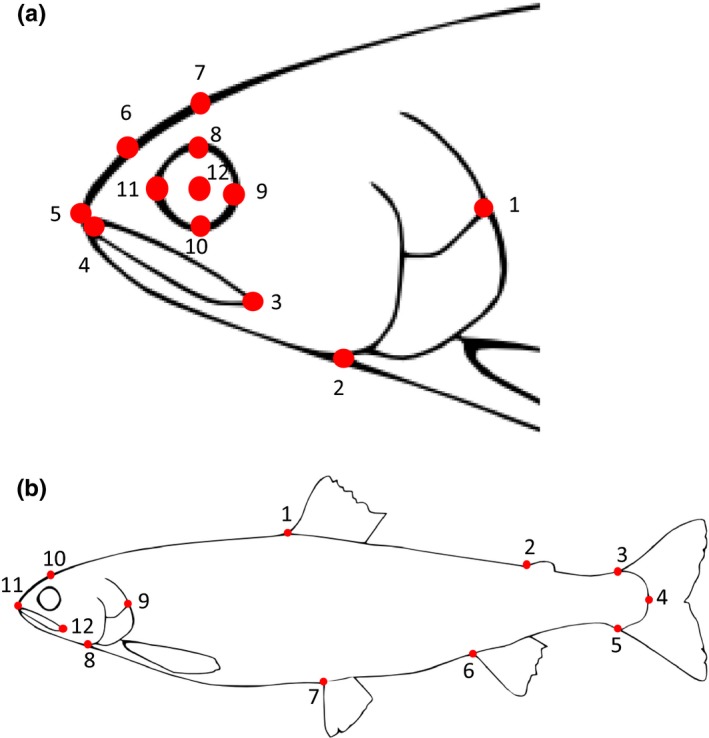
Placement of landmarks for body and head shape analyses. For description of the landmarks, see Table S1 (Appendix S1). Drawing: Sigrid Skoglund

For the morphological analyses, the left side of each fish was photographed from a distance of 60 cm using a digital camera (Nikon Coolpix 5400) under standard light conditions. Each fish was carefully flattened laterally and attached to a polystyrene plate with dissecting pins before being photographed (Frederich & Sheets, 2010; Muir, Vecsei, & Krueger, 2012). Each individual was only photographed once, thus not allowing to test for any posture‐related variation in body shape. The photographs were imported to tpsUtil v. 1.5.3 (Rohlf, 2010b) and then opened in tpsDig v. 2.16 (Rohlf, 2010a) for the placement of landmarks (Figure 3). Head and body shape were analyzed separately, and 12 landmarks were used for each analysis (Figure 3, Table S1 of Appendix S1). To reduce measurement errors, the same person performed the landmark placement on all fish (Frederich & Sheets, 2010) and the same camera, camera lens, photographic setups, scale bars, and tripod were used (Arnqvist & Martensson, 1998; Collins & Gazley, 2017). In accordance with prevailing literature on preventing measurement errors, we used a crop‐sensor camera with more than five megapixels (Collins & Gazley, 2017; Muir et al., 2012).

### 2.5 Statistical methods

To remove nonshape effects from the landmark coordinates, a Procrustes superimposition was performed in MorphoJ (Klingenberg, 2011), standardizing position, scale, and orientation of the specimens. The resulting Procrustes shape coordinates were used in Principal Component Analyses (PCAs) to identify the shape variables that captured most of the morphological variation (Mitteroecker & Gunz, 2009). The principal components are variables that are orthogonal, uncorrelated, with the data. PCAs were performed separately on body and head shape in MorphoJ. The effects of size, morph, and their interaction on derived PC scores were analyzed using, respectively, MANCOVAs and thereafter ANCOVAs (type II) on the first five PC axes in the [R] team package “car” (Fox & Weisberg, 2011). The log value of centroid size (logCS) was used as a measure of body or head size. Centroid size was computed as the square root of the summed squared deviations of the coordinates (Mitteroecker & Gunz, 2009). Pairwise post hoc tests were performed on the ANCOVAs for detection of which morphs differed from each other when there was a morph effect or a size*morph interaction effect, and pairwise tests were subject to Bonferroni's corrections. Within each morph, relationships between PC scores and size were explored using linear regression. Head shape variation with size gave similar statistical results using body or head size (logCS) as the size measure, and the results using head size are presented here. We used a change in shape with size as the measure of ontogenetic change because size relates directly to growth, whereas time may not (Boughton, Collette, & McCune, 1991; Strauss, 1987).

To explore allometric trajectories between morphs, we looked at significant results for the morph and morph*size interaction effects (Table 1) from the pairwise post hoc tests described above. A significant interaction effect was interpreted as the morph pair being on different allometric trajectories (Svanbäck & Eklöv, 2002) either convergent or divergent. To decide whether trajectories were convergent or divergent, the slopes of the linear regressions were examined. When there was no interaction effect and no morph effect, the morphs were on common allometric trajectories, whereas if there was a morph effect but no interaction effect there were parallel trajectories.

**Table 1:** Type of allometric trajectories for the morph pairs based on significant morph effects and/or morph*size interaction effects in ANCOVA models of shape with size and morph as explaining variables

Morph effect	Morph*size interaction	Trajectory
No	No	Common trajectory
No or yes	Yes	Convergent or divergent trajectories
Yes	No	Parallel trajectories

Convergent and divergent trajectories can be differentiated based on the slopes of morph‐specific linear regressions.

Sex differences were explored, but gender only turned out to be a significant variable for the LO‐morph for PC1 body shape (*p* = .0006). Based on this, we pooled the sexes for the analyses. Regarding maturation, we included both mature and immature fish in our dataset, and fish were captured throughout the autumn and early winter, including the spawning season of the LO‐morph. Because of this, we chose to not include landmarks on the anterior side of the belly, as this part of the body would most likely be affected by maturation status (near spawning or not). However, first onset of maturation is indirectly included in our study as we include the size of the fish (logCS) as a variable, and most often there is a clear relationship between size of the fish and maturation for each morph.

## 3 RESULTS

### 3.1 Morph classification

#### 3.1.1 Phenotypic classification

The numbers of fish kept for the following morphological analyses were 60 PB‐morph, 92 PP‐morph, and 97 LO‐morph. The PB‐morph were 7.3–15.1 cm FL (mean: 11.4 cm ± SD: 1.8), the PP‐morph were 9.9–44.8 cm FL (mean: 22.4 cm ± SD: 7.9), and the LO‐morph were 9.1–46.8 cm FL (mean: 20.5 cm ± SD: 7.8).

#### 3.1.2 Genotypic classification

The STRUCTURE analysis confirmed that all three phenotypically divergent charr morphs also can be discriminated genetically (*K* = 3; ln Pr(*Χ*|*Κ*) = −3,109.2 ± 0.7, Δ*K* = 614, Appendix S3, Table S5). Most individuals displayed high membership coefficients to the assigned clusters, except for three adult PB‐morphs and five adult PP‐morphs that had membership coefficients between 0.714 and 0.879 (Appendix S3). These eight individuals were excluded from further analysis.

The assignment of the other ontogenetic life stages to the adult reference populations revealed high mean assignment scores (LO = 100.0%; PB = 97.8%; PP = 99.8%). Similar high assignment success was observed using probability testing and STRUCTURE analysis (Appendix S3). The three approaches consistently assigned each individual to one of the three reference populations, except for five individuals, corresponding to 4.2% of the assigned individuals, where two of the three approaches where in agreement. However, the genetic assignment was supported by the phenotypic classification for four of these five individuals (Appendix S3). The fifth individual (Skg11387, in Appendix S3, Table S6) appeared to be a hybrid of the PB‐ and PP‐morphs, but was included as a PP‐morph based on its diet and growth. This individual had eaten six‐three‐spined sticklebacks (piscivory) and the growth curve corresponded to the growth curve of the PP‐morph (Smalås et al., 2013) making the individual functionally and ecologically acting as a PP‐morph.

#### 3.1.3 Consensus of the morph classification

There was compliance between the phenotypic classification and the genetic assignment as the two approaches were in agreement for 96%, 96%, and 100% of the LO‐, PB‐, and PP‐morphs, respectively (Appendix S3). The two individuals (Skg11387, mentioned above, and Skg11387, Appendix S3) that constitutes the 4% disagreement between the approaches for the LO‐ and PB‐morphs, where included in the shape analyses based on the phenotypic classification.

### 3.2 Body shape

Using MANCOVA, it was found significant size, morph, and morph*size interaction effects (Table 2) on body shape. The ANCOVAs showed that in total, PC‐axes 1–5 explained 81.4% of the observed body shape variance. There were significant overall size effects in three of the first five PC‐axes, two of the five PC‐axes showed a significant morph*size interaction effect, and there were significant morph effects in four of the five‐first PC‐axes (Table 3). The second PC‐axis showed an unwanted lunate‐like distortion of the fish and is not considered further since this is not biologically relevant (Fig. S1 of Appendix S1). The main emphasis when exploring allometric changes in body shape will be on PC‐axis 1 and 3 that showed most of the remaining explained variance. The results from the remaining PC‐axes are shown in Fig. S1 and Tables S2, S3 in Appendix S1.

**Table 2:** Effects of size (log centroid size), morph, and their interaction on body and head shape using MANCOVAs, test Pillail

	Size		Morph		Morph*size	
	Pillai1;243	*p* value	Pillai2;243	*p* value	Pillai2;243	*p* value
Body	0.73	**<.0001**	1.62	**<.0001**	0.50	**<.0001**
Head	0.82	**<.0001**	1.56	**<.0001**	0.51	**<.0001**

Significant *p* values (<.05) are indicated by boldface.

**Table 3:** Effects of size (log centroid size), morph, and their interaction, on the first five PC‐axes (% variance explained) for body and head shape using ANCOVAs

	Size			Morph			Morph*size			Resid.
	*F*_1;243_	*p* value	Var.	*F*_2;243_	*p* value	Var.	*F*_2;243_	*p* value	Var.	Var.
Body										
PC1 (39.8%)	3.0	.0844	0.3	378.5	**<.0001**	75.2	1.3	.2628	0.3	24.2
PC2 (18.3%)	15.0	**.0001**	5.7	3.5	**.0310**	2.6	0.6	.5317	0.6	91.1
PC3 (11.7%)	226.1	**<.0001**	40.9	34.3	**<.0001**	12.3	7.6	**.0006**	2.6	44.1
PC4 (6.4%)	0.2	.6501	0.1	7.7	**.0006**	5.7	1.4	.2597	1.1	93.1
PC5 (5.3%)	11.2	**.0010**	4.2	2.5	.0817	2.1	8.8	**.0002**	6.3	87.3
Head										
PC1 (31.6%)	161.1	**<.0001**	35.8	17.8	**<.0001**	7.9	5.1	**.0066**	2.3	54.0
PC2 (23.9%)	81.1	**<.0001**	18.8	53.1	**<.0001**	24.6	0.5	.6200	0.2	56.3
PC3 (12.0%)	10.6	**.0013**	2.6	70.3	**<.0001**	34.4	7.3	**.0008**	3.6	59.5
PC4 (7.4%)	24.1	**<.0001**	7.7	20.1	**<.0001**	12.7	4.0	**.0206**	2.5	77.1
PC5 (6.1%)	3.0	.0864	1.1	9.5	**.0001**	6.8	5.6	**.0042**	4.1	88.0

Significant *p* values (<.05) are indicated by boldface. Var. = % variance explained by the sum of squares.

**Figure 4:**
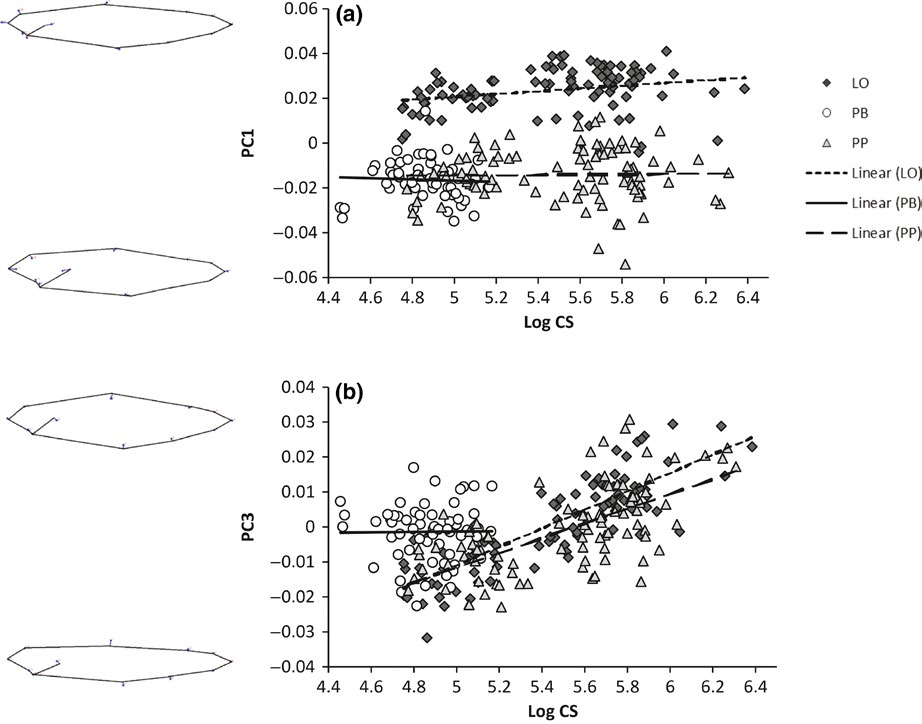
Body shape changes through growth of the three morphs in Lake Skogsfjordvatnet. PC scores are plotted against Log centroid body size. (a) PC1 (b) PC3. Body shapes at extreme values on each PC‐axis are illustrated by wireframe drawings

PC‐axis 1 mainly described differences in head size and positioning of the dorsal and pelvic fins (Figure 4a). Fish with a high score for PC1 had a smaller head, and the dorsal and pelvic fins were placed more to the anterior end of their body. Across all morphs, the effect of size on PC1 scores was just outside statistical significance (*p* = .0844, Table 3) suggesting that there may be some size effect in one or more individual morphs. Thus, this was tested further using post hoc morph‐specific Bonferroni's corrected regressions. These showed that the LO‐morph had significantly higher PC1 scores with increasing size of the fish although the magnitude of change with size was small (Table 4, Figure 4a). There was no interaction between morph and size, indicating that all morphs had the same allometric shape size relationship, but there was a significant overall difference between the morphs (Table 3). The PP‐ and PB‐morph were similar in the expression of shape traits described by PC‐axis 1, while the LO‐morph had significantly higher PC scores than the other two morphs (Table 4, Figure 4a). Throughout their entire size range, the PP‐ and PB‐morph had a relatively bigger head than the LO‐morph. The positioning of the dorsal and pelvic fins was more to the anterior end of the body for the LO‐morph than for the other two morphs.

**Table 4:** Results from linear regressions within each morph, testing for size effects in PC1 and PC3 of body shape and PC1 and PC2 of head shape

	LO		PP		PB	
	*F*_1;95_	*p* value	*F*_1;90_	*p* value	*F*_1;58_	*p* value
Body						
PC1	6.6	**.0120**	0.0	.8651	0.2	.6909
PC3	208.9	**<.0001**	67.5	**<.0001**	0.0	.9299
Head						
PC1	42.5	**<.0001**	119.8	**<.0001**	12.2	**.0009**
PC2	35.5	**<.0001**	40.1	**<.0001**	5.6	**.0217**

Significant *p* values (<.05) are indicated by boldface.

The LO‐morph was on a parallel trajectory in body shape PC1 to both the PP‐ and PB‐morph (Table 5, Figure 4a). The PP‐ and PB‐morph were on a common allometric trajectory (Table 5, Figure 4b).

**Table 5:** Pairwise ANCOVAs for PC1 and PC3 of body shape and PC1 and PC2 of head shape, exploring effects of size (log centroid size), morph, and morph*size interaction

	*df*	Size		Morph		Morph*size		All. traj.
		*F*	*p* value	*F*	*p* value	*F*	*p* value	
Body								
PC1								
LO‐PP	1;185	3.2	.0753	579.5	**<.0001**	1.9	.1658	Parallel
LO‐PB	1;153	5.7	.0184	331.2	**<.0001**	1.4	.2406	Parallel
PB‐PP	1;148	0.0	.9451	0.7	.4006	0.1	.7126	Common
PC3								
LO‐PP	1;185	239.1	**<.0001**	7.5	**.0066**	2.8	.0938	Parallel
LO‐PB	1;153	167.4	**<.0001**	47.3	**<.0001**	16.9	**.0001**	Convergent
PB‐PP	1;148	65.6	**<.0001**	28.9	**<.0001**	7.7	**.0062**	Convergent
Head								
PC1								
LO‐PP	1;185	151.1	**<.0001**	20.2	**<.0001**	10.4	**.0015**	Conv./div.
LO‐PB	1;153	52.9	**<.0001**	30.8	**<.0001**	1.2	.2849	Parallel
PB‐PP	1;148	128.3	**<.0001**	0.8	.3849	0.3	.6058	Common
PC2								
LO‐PP	1;185	75.6	**<.0001**	90.8	**<.0001**	1.0	.3291	Parallel
LO‐PB	1;153	39.5	**<.0001**	32.9	**<.0001**	0.1	.7806	Parallel
PB‐PP	1;148	47.3	**<.0001**	1.8	.1830	0.1	.8303	Common

All. traj, allometric trajectory; conv., convergent; div, divergent.

Significant *p* values after Bonferroni's correction (<.0167) are indicated by boldface.

PC‐axis 3 mainly described a change in body height (Figure 4b). Fish with a high score on PC3 had a greater body height than fish with a lower score. There was an overall effect of fish size on PC3 (Table 3), the bigger the fish, the greater the body height, indicating that across all morphs combined there was a significant allometric effect on this shape measure. Linear models for the separate morphs revealed that this allometric effect was significant for the LO‐ and PP‐morph, but not for the PB‐morph (Table 4). There was a significant effect of the morph*size interaction (Table 3), and the pairwise tests showed that the allometric shape change was different in the PB‐morph compared to the two other morphs, which were similar (Table 5). There was also a significant overall difference between the morphs (Table 3), and all the three morphs differed from each other (Table 5). The PB‐morph had higher PC‐scores than the other two morphs for fish of the same size (Figure 4b). This means that the PB‐morph in general had a relatively greater body height for a given body size. The LO‐morph showed higher PC‐scores, and thus greater body height, than the PP‐morph of similar length (Figure 4b).

The PB‐morph was on convergent trajectories in body shape PC3 to both the LO‐ and PP‐morph (Table 5, Figure 4b). The LO‐ and PP‐morph were on parallel trajectories (Table 5, Figure 4b).

### 3.3 Head shape

The MANCOVA for head shape showed significant size, morph and morph*size interaction effects (Table 2). The ANCOVAs showed that PC‐axes 1–5 explained 80.9% of the variance of head shape. The ANCOVAs of the first five PC‐axes revealed that all five showed significant morph effects, four showed significant size effects and four showed a morph*size interaction effect (Table 3). The focus here will be on PC‐axes 1 and 2 as they explain most of the variance (31.6% and 23.9%, respectively). The results for the remaining PC‐axes are given in the Appendix S1 (Fig. S2, Tables S2, S3).

**Figure 5:**
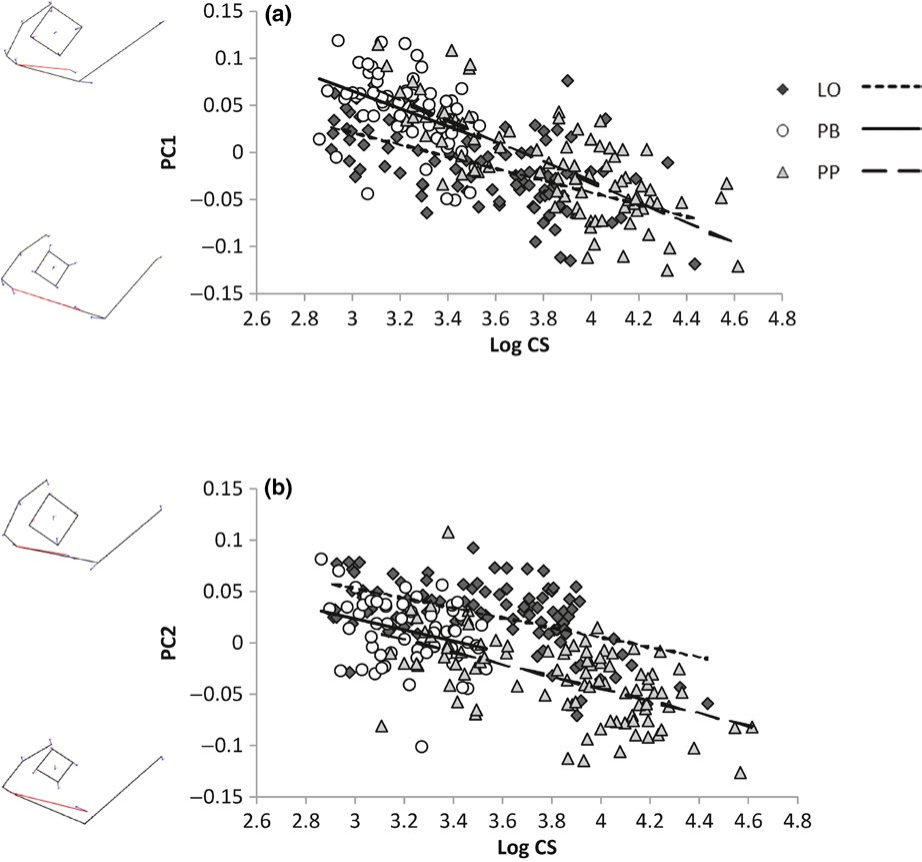
Head shape changes through growth of the three morphs in Lake Skogsfjordvatnet. PC scores are plotted against Log centroid head size. (a) PC1 (b) PC2. Head shapes at extreme values on each PC‐axis are illustrated by wireframe drawings

PC‐axis 1 mainly described the size of the eye and the mouth (Figure 5a). A high score for PC1 described a fish with a large eye and a small mouth. Along PC‐axis 1, there was a significant overall change in shape with size (Table 3), and linear models showed that this was also significant for all the individual morphs (Table 4). All three morphs developed a relatively larger mouth and smaller eyes as they increased in size (Figure 5a). Pairwise tests showed that the LO‐ and PP‐morph had significantly different trajectories for PC1 (Table 5). The increase in mouth size and decrease in eye size was relatively faster for the PP‐morph compared to the LO‐morph (Figure 5a). Pairwise tests for morph effects showed that the LO‐morph was significantly different from the PP‐ and PB‐morph (Table 5). The LO‐morph had relatively smaller eyes and larger mouth than the other two morphs at the smallest sizes included in this study.

The PP‐ and PB‐morph were on a common allometric trajectory for head shape PC1, and the PB‐ and LO‐morph were on parallel trajectories (Table 5, Figure 5a). The LO‐ and PP‐morph were on mainly converging trajectories up to a logCS head size of approx. 4.2 thereafter diverging (Table 5, Figure 5a).

PC‐axis 2 mainly described the length of the mouth and the depth of the head (Figure 5b). Fish with a high PC score on PC‐axis 2 had a short mouth and a less deep head. There was an overall size effect for PC‐axis 2 (Table 3), and linear models showed that the size effect also applied to all the morphs separately (Table 4). When they were small, fish from all morphs had a short mouth and a less deep head. There was no morph*size interaction effect, but the LO‐morph was significantly different from the two other morphs (Table 3, 5). The LO‐morph had a shorter mouth and a less deep head compared to the PP‐ and PB‐morph of the same size.

The LO‐morph was on a parallel trajectory to the PP‐ and PB‐morph for head shape PC2, while the PP‐ and PB‐morph were on a common trajectory (Table 5, Figure 5b).

## 4 DISCUSSION

Our morphological studies demonstrate distinct differences in the allometric trajectories between the three genetically differentiated Arctic charr morphs in Lake Skogsfjordvatn. We observed allometric scaling (i.e., a change in morphology with size) in several of the studied PC axes, and the three morphs showed clear evidence of differences in allometry, reflecting different growth trajectories. Patterns of common, parallel, and convergent allometric trajectories were all observed in the pairwise comparisons of the morphs. More specifically, the two profundal morphs (the PB‐ and PP‐morphs) shared common trajectories for variables explaining most of their body and head shape variation. Thus, PC1 body shape and PC1 and PC2 head shape all indicate common allometric trajectories that conform to model d (Figure 1). This indicates that the selection pressure has relatively low impact on divergence in the allometric trajectories and relatively low inherited divergence in head or body shape between these two profundal morphs. Rather morphological differences between the PP‐ and PB‐morphs are the result of a termination of growth at relatively small size in the PB‐morph compared with the PP‐morph, which continues to grow (Smalås et al., 2013). This pattern suggests that selection pressures on the two morphs are similar at least through the size range expressed by the PB‐morph. It also suggests that both morphs inherit similar allometric processes that are not modified differentially by plasticity. This supports our first prediction and is likely a result of a common habitat and diet utilization of the morphs in their overlapping size range (Knudsen et al., 2016).

In contrast to the PP‐PB trajectory comparisons, the LO‐PP and LO‐PB comparisons conformed to allometric trajectories described by models a and c (Figure 1). Body shape and head shape differences between both morph pairs were best explained by a parallel process (a common allometry) with different trajectory origins (model a, Figure 1). Thus, the LO‐PP body shape (PC1) and head shape (PC2) comparisons and LO‐PB body shape (PC1) and head shape (PC1 and PC2) showed parallel allometric relationships. The LO‐PP morph comparison of head shape (PC1) in contrast showed evidence of convergent growth processes (model c, Figure 1). Converging allometry in morphological traits is found among several different related species or morphs from many vertebrates (Grundler & Rabosky, 2014; Santana & Cheung, 2016; Winemiller, Kelso‐Winemiller, & Brenkert, 1995). Converging morphology is indicating common functional adaptations among the individuals or groups being studied (Losos, 2011). Convergence enables optimal solutions to problems repeatedly posed by the environment to be “solved” by natural selection. The morphological adaptations can be related to a converging diet or habitat use, for example, found for lizards (Scleroglossa and Iguania; Losos, Jackman, Larson, de Queiroz, & Rodríguez‐Schettino, 1998; Stayton & Schwenk, 2006). For herbivorous lizards, there is a converging morphology in skulls and lower jaws, probably an adaptation directly related to a common diet, thereby diverging herbivores from their carnivorous relatives (Stayton & Schwenk, 2006). For the LO‐PP morph comparison, the convergence in head shape (PC1) is probably also related to their diet. The allometric pathways are different but the largest fish of the two morphs are quite similar due to the convergence.

The often parallel trajectories of the LO‐PP morph and the LO‐PB morph may be a result of mostly similar growth processes acting upon different trajectory starting points. This indicates that selection has low impact on divergence of the allometric trajectory of body and head shape on body size, rather that morph differences are a result of different starting points (the size of the fish when they entered this study), for both the LO‐PP morph and LO‐PB morph. Differences in head and body morphology between littoral and profundal morph pairs have earlier been found to be inherited from experimental studies (Klemetsen, Elliott, Knudsen, & Sørensen, 2002). Maybe the different trajectory starting points of the morphs in this study have resulted from inherited differences in head and body shape, which could be genetic in origin or the result of some maternal effect. Parallel ontogenetic trajectories, probably occurred prenatally showing parallel trajectories after birth, are found for cranial morphology in primates (Mitteroecker et al., 2004). The parallel trajectories for the LO‐PP and LO‐PB morph comparisons can also have evolved prenatally or at very early life stages based on strong selection pressures early in life due to different environmental conditions in their respective habitats. The time of onset of divergence between the morphs should be investigated in the future studies.

The differences in body shape and head morphology expressed in these morphs are very likely to have functional consequences. In our results for head shape, we recognize traits that probably can be both “life‐or‐death” (predator avoidance) and food‐gathering traits (Koehl, 1996). For the PB‐morph, greater body depth may constitute an important adaptation to the predation threat from the PP‐morph as this trait is known to reduce the predation vulnerability in fish (Brönmark & Miner, 1992; Persson, Andersson, Wahlström, & Eklöv, 1996). The consistently deep body of the profundal PB‐morph may be also partly related to its predominantly benthic diet (Amundsen, Knudsen, & Klemetsen, 2008; Knudsen, Klemetsen, Amundsen, & Hermansen, 2006) as this feature is typical of other benthivore fish morphs (Knudsen et al., 2011; Robinson & Parsons, 2002; Schluter & Nagel, 1995; Svanbäck & Eklöv, 2002). The PP‐morph was more streamlined in body shape than the other two morphs, probably reflecting the PP‐morph`s piscivorous specialization that favors a fusiform body shape (Webb, 1984). Furthermore, the fin placement of the morphs also differs with the PP‐ and PB‐morphs having a more posterior placement of fins then the LO‐morph. This fin format is known to increase thrust which would facilitate piscivory in sit and wait predators (Webb, 1977). In contrast, the more caudal positioned dorsal fin in the PB‐morph could be a response to selection for burst swimming in order to escape predators (Scharnweber et al., 2013).

Head size relative to body size varied between morphs. The large heads relative to body size for the PP‐ and PB‐morph throughout their entire size range suggest that they can eat relatively larger prey all of their life compared with the LO‐morph (Parsons, Skúlason, & Ferguson, 2010; Snorrason et al., 1994). The relatively big head of the PP‐morph is likely to be an adaptation to piscivore feeding, where large food item size means that a larger head (and thus gape) allows them to start feeding on fish relatively early in life (Knudsen et al., 2016). Other piscivore animals can have other adaptations to their diets, for example, bats (Mammalia; Chiroptera) may develop very long wings giving low flight power and cost of transport when foraging fish over open stretches of water (Norberg & Rayner, 1987). For the PB‐morph, the relative large head may be a result of paedomorphism (developmental heterochrony) during their early life stages (Alekseyev et al., 2014; Skúlason, Noakes, & Snorrason, 1989), making the PB‐morph retain its juvenile head characteristics. The relatively smaller head size in the LO‐morph (compared to the PP‐ and PB‐morph) probably reflects the omnivorous nature of the diet of this form which mostly comprise prey of smaller particle size (i.e., zooplankton) than that taken by the PP‐morph (Skoglund, Knudsen, & Amundsen, 2013; Smalås et al., 2013). Adaptation and constraints related to diet and morphology are found among ecologically comparable fishes across the globe (Davis, Pusey, & Pearson, 2012; Winemiller et al., 1995).

Big eyes relative to head size in the PB‐ and PP‐morph that both reside in the dark profundal zone are likely connected to visual acuity in low light related to food gathering (Alexander, 1987). Larger eyes may also help to avoid predation. Snorrason et al. (1994), for example, considered big eye size in juveniles vulnerable to predation, to be an anti‐predation trait in all four Arctic charr morphs from Thingvallavatn. That same study in addition found (as was shown here) that the eyes of the four Arctic charr morphs tended to become relatively smaller as the fish grew larger. Deep‐water morphs of whitefish have also been found to have relatively big eyes (Siwertsson, Knudsen, Adams, Præbel, & Amundsen, 2013) as also found in deep‐water adapted taxa living in the sea (Warrant & Locket, 2004).

Here we show that the PP‐ and PB‐morphs were on a common allometric trajectory for head shape traits mostly related to the length of the mouth and the depth of the head. In contrast, the LO‐morph was on a parallel allometric trajectory to the two deep‐water morphs with a less deep head and a shorter mouth. Different starting points of the trajectories suggest different selective pressures on the PP‐ and PB‐morphs compared to the LO‐morph at the early life stages (Klingenberg, 1998) resulting from either inherited differences (genetic or through maternal influence) or from differential processes in very early ontogeny (at sizes smaller than examined in this study). There was a size effect for all the morphs with the head getting deeper and with a longer mouth with increasing size of the fish, suggesting that the selective pressure operating to drive allometric changes is common for all morphs. For example, a deeper head is probably related to the feeding of successively bigger prey for all morphs (Parsons et al., 2010; Snorrason et al., 1994).

The allometric patterns of both head and body shape change with the size point toward different levels of divergence between the morph pairs. The PP‐ and PB‐morphs share both a common trajectory starting point (at the size when they enter this study) and identical allometry for the main head and body shape variables. This points to a close origin for this morph pair, with the morphology of the PB‐morph arising from a cessation in the increase in body size (paedomorphism) compared with the PP‐morph. Similar common ontogenetic trajectories have been found for *Podarcis* lizards (Piras et al., 2011). For these lizards, there is a prolongation of growth along the same trajectories that is producing both intersexual and interspecific morphological differences through the process of hypermorphosis. Rapid phenotypic diversification can be facilitated through the process of hypermorphosis, enabling functional and ecological relevant traits to be generated quickly. One possibility is that the PP‐morph has evolved by hypermorphic processes from the PB‐morph, creating a morph utilizing a piscivore niche in the profundal zone. In contrast, the evidence of this study is that the allometric processes that give rise to the LO‐morph differ substantively from that of the PP‐ or PB‐morph. With the LO‐morph differing in allometric origin for all the main body and head morphological differences, this strongly points to an inherited (genetically or through maternal effects) difference in shape between the LO‐morph and the other two morphs (Klemetsen et al., 2002). This indicates that the LO‐morph is more diverged from the PP‐ and PB‐morphs than the PP‐ and PB‐morphs are from each other. However, this was not reflected by the genetic analyses performed herein (Appendix S3, Table S7). The LO‐morph in Lake Skogsfjordvatn was less diverged genetically from the PP‐ and PB‐morphs than the PP‐ and PB‐morphs were from each other, suggesting that the divergence in allometric processes follows another genetic trajectory than neutral genetic divergence. This is most likely because of similar adaptation to the deep‐water environment of the PB‐ and PP‐ morphs related to morphological adaptations to low temperature and light conditions (Luk et al., 2016; Warrant & Locket, 2004) and to less need for maneuverability in a less complex habitat structure.

In conclusion, the three predictions outlined are supported for some of the variance in head or body shape. Common allometric trajectories occurred between the PP‐ and PB‐morph for three of the four PC‐axes examined, which is likely a result of a recent common evolutionary history and similar habitat and diet utilization of similar‐sized individuals. Parallel trajectories occurred between both the LO‐ and PB‐morph and the LO‐ and PP‐morph for several of the PC‐axes implying that the selection processes that have induced the differentiated morphologies are operating at earlier life stages (Sheets & Zelditch, 2013). Convergent allometric trajectories were only found once among all the morph pairs. Convergent trajectories suggest that there are similar functional demands on these traits at larger fish size despite inherited trait differences (Adams & Nistri, 2010).

## CONFLICT OF INTEREST

None declared.
