# Supplementary material for: Allometric trajectories of body and head morphology in three sympatric Arctic charr (Salvelinus alpinus (L.)) morphs
Source: Ecol Evol. 2017 Aug 8;7(18):7277–89. doi: 10.1002/ece3.3224 (PMC5606865; doi:10.1002/ece3.3224)
Supplement: Supplementary file 1 [file ECE3-7-7277-s001.docx]

**Electronic supplementary**

**Appendix S1**

Table S1: Description of the position of the 12 landmarks for body and head shape.

|  | **LM** | **Position** |
| --- | --- | --- |
| **Body** | 1 | Anterior insertion of dorsal fin |
|  | 2 | Anterior insertion of adipose fin |
|  | 3 | Dorsal insertion of caudal fin |
|  | 4 | Midpoint of hypural plate |
|  | 5 | Ventral insertion of caudal fin |
|  | 6 | Anterior insertion of anal fin |
|  | 7 | Anterior insertion of pelvic fin |
|  | 8 | Ventral margin of opercular bone |
|  | 9 | Posterior end of intersection between operculum and suboperculum |
|  | 10 | Top of cranium directly above eye |
|  | 11 | Anterior tip of snout |
|  | 12 | Posterior tip of maxilla |
| **Head** | 1 | Anterior end of intersection between operculum and suboperculum |
|  | 2 | Ventral margin of opercular bone |
|  | 3 | Posterior tip of maxilla |
|  | 4 | Anterior tip of maxilla |
|  | 5 | Anterior tip of snout |
|  | 6 | Midway between LM 5 and LM 7 |
|  | 7 | Top of cranium directly above eye |
|  | 8 | Superior margin of the eye |
|  | 9 | Posterior margin of the eye |
|  | 10 | Inferior margin of the eye |
|  | 11 | Anterior margin of the eye |
|  | 12 | Center of the eye |

**
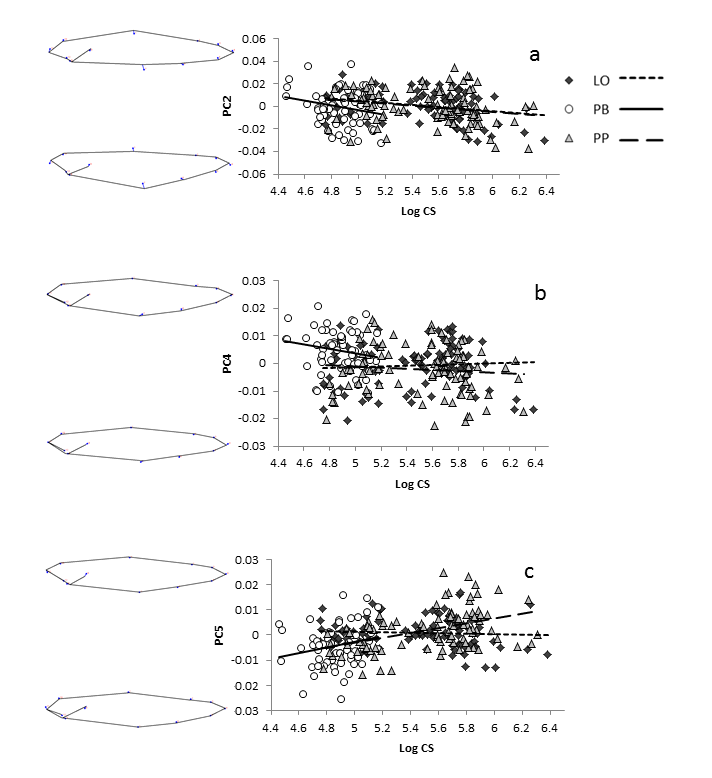
**

Fig. S1: Body shape changes through growth of the three morphs in Lake Skogsfjordvatnet. PC scores are plotted against Log centroid body size. A) PC2 B) PC4 C) PC5. Body shapes at extreme values on each PC-axis are illustrated by wireframe drawings.


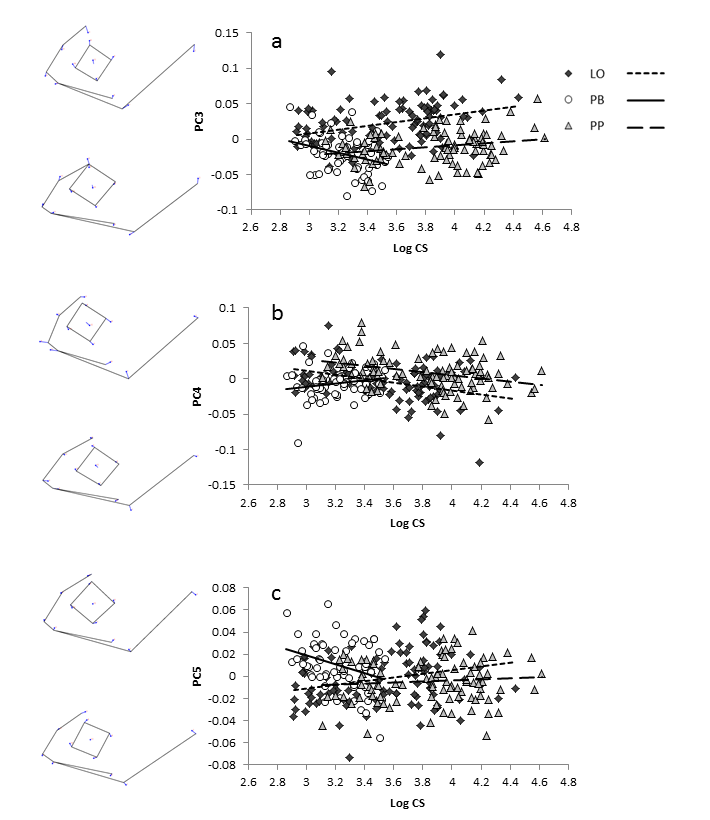


Fig. S2: Head shape changes through growth of the three morphs in Lake Skogsfjordvatnet. PC scores are plotted against Log centroid head size. A) PC3 B) PC4 C) PC5. Head shapes at extreme values on each PC-axis are illustrated by wireframe drawings.

Table S2: Linear regressions testing for size effects on shape for each of the three morphs. p-values < 0.0500 are regarded as significant and indicated by boldface.

|  |  | **LO** |  | **PP** |  | **PB** |  |
| --- | --- | --- | --- | --- | --- | --- | --- |
|  |  | **F_1;95_** | **p-value** | **F_1;90_** | **p-value** | **F_1;58_** | **p-value** |
| **Body** | **PC2** | 9.1 | **0.0034** | 5.0 | **0.0282** | 3.2 | 0.0788 |
|  | **PC4** | 0.4 | 0.5151 | 0.7 | 0.3947 | 1.9 | 0.1706 |
|  | **PC5** | 0.4 | 0.5349 | 23.0 | **<0.0001** | 3.1 | 0.0838 |
| **Head** | **PC3** | 12.6 | **0.0006** | 4.4 | **0.0389** | 6.8 | **0.0117** |
|  | **PC4** | 16.5 | **0.0001** | 11.2 | **0.0012** | 2.5 | 0.1187 |
|  | **PC5** | 6.1 | **0.0156** | 0.8 | 0.3841 | 6.3 | **0.0149** |

Table S3: Pairwise ANCOVAs for PC2, PC4 and PC5 body shape and PC3-PC5 head shape. p-values < 0.0167 (Bonferroni corrections) are regarded as significant and indicated by boldface.

|  |  |  |  | **Size** |  | **Morph** | | **Morph*size** | |
| --- | --- | --- | --- | --- | --- | --- | --- | --- | --- |
|  |  |  | **df** | **F** | **p-value** | **F** | **p-value** | **F** | **p-value** |
| **Body** | **PC2** | **LO-PP** | 1;185 | 13.3 | **0.0003** | 0.1 | 0.8208 | 0.0 | 0.9551 |
|  |  | **LO-PB** | 1;153 | 10.2 | **0.0017** | 6.3 | **0.0128** | 1.3 | 0.2642 |
|  |  | **PB-PP** | 1;148 | 7.1 | **0.0084** | 3.9 | 0.0501 | 1.1 | 0.3066 |
|  | **PC4** | **LO-PP** | 1;185 | 0.0 | 0.8786 | 1.5 | 0.2250 | 1.2 | 0.2810 |
|  |  | **LO-PB** | 1;153 | 0.0 | 0.8342 | 11.2 | **0.0010** | 2.2 | 0.1372 |
|  |  | **PB-PP** | 1;148 | 1.6 | 0.2058 | 6.7 | **0.0105** | 0.8 | 0.3709 |
|  | **PC5** | **LO-PP** | 1;185 | 9.8 | **0.0021** | 1.0 | 0.3089 | 17.9 | **<0.0001** |
|  |  | **LO-PB** | 1;153 | 0.0 | 0.8989 | 9.7 | **0.0022** | 4.6 | 0.0337 |
|  |  | **PB-PP** | 1;148 | 24.3 | **<0.0001** | 0.0 | 0.8397 | 0.1 | 0.7957 |
| **Head** | **PC3** | **LO-PP** | 1;185 | 16.6 | **<0.0001** | 103.9 | **<0.0001** | 1.8 | 0.1803 |
|  |  | **LO-PB** | 1;153 | 6.5 | **0.0118** | 61.0 | **<0.0001** | 13.0 | **0.0004** |
|  |  | **PB-PP** | 1;148 | 0.8 | 0.3843 | 1.3 | 0.2626 | 11.0 | **0.0012** |
|  | **PC4** | **LO-PP** | 1;185 | 27.6 | **<0.0001** | 27.2 | **<0.0001** | 0.3 | 0.5641 |
|  |  | **LO-PB** | 1;153 | 12.8 | **0.0005** | 4.6 | 0.0327 | 8.0 | **0.0054** |
|  |  | **PB-PP** | 1;148 | 7.7 | **0.0061** | 22.5 | **<0.0001** | 6.8 | **0.0100** |
|  | **PC5** | **LO-PP** | 1;185 | 6.1 | **0.0146** | 2.9 | 0.0923 | 1.7 | 0.1909 |
|  |  | **LO-PB** | 1;153 | 2.3 | 0.1280 | 11.9 | **0.0007** | 9.8 | **0.0021** |
|  |  | **PB-PP** | 1;148 | 0.0 | 0.8526 | 8.1 | **0.0051** | 7.9 | **0.0057** |

**Appendix S2 (genetics).**

**Amplification and validation of the microsatellite loci used for the genetic classification of charr morphs**

**Materials and Methods**

*Microsatellite amplification and scoring*

DNA was extracted using the E-Z96 Tissue DNA Kit (OMEGA Bio-tek®) following the manufacturer’s protocol. Nine microsatellite loci were arranged in two multiplex panels (Table S4) and amplified using the polymerase chain reaction (PCR) on a GeneAmp 9700 thermal cycler (Applied Biosystems). Each reaction consisted of 1.25 µl QIAGEN® Multiplex PCR Master Mix, 0.25 µl primer mix (multiplex 1 or 2), 0.5 µl water and 5-10 ng template DNA. The PCR profile for multiplex panel 1 was: 95°C for 15 min followed by 26 cycles of 94°C for 30 s, 60°C for 3 min and 72°C for 1 min, with a final 60°C extension for 30 min. The PCR profile for multiplex panel 2 was: 95°C for 15 min followed by 26 cycles of 94°C for 30 s, 55°C for 3 min and 72°C for 1 min, with a final 60°C extension for 30 min. The PCR products were separated on an ABI 3130XL Automated Genetic Analyzer (Applied Biosystems) using GeneScan™ 500 LIZ® Size Standard (Applied Biosystems) as internal standard. The alleles were automatically binned in predefined allelic bins in the GeneMapper 3.7 software (Applied Biosystems) and verified by visual inspection.

*Validation of the microsatellite loci*

The software MICRO-CHECKER 2.2.3 (Van Oosterhout et al. 2004) was used to screen the samples for abnormalities (null alleles, scoring errors, etc.) and 1,000 bootstraps were used to generate the expected homozygote and heterozygote allele size difference frequencies. Departures from Hardy-Weinberg equilibrium (HWE) for each locus and possible linkage disequilibrium (LD) between loci were estimated by exact tests (Guo and Thompson 1992) in GENEPOP 4.0 (Rousset 2007). P-values for these estimates are presented unadjusted and adjusted for multiple comparisons (type I errors) using sequential Bonferroni corrections (Rice 1989).

**Results**

The locus Sco215 was monomorphic in all populations and was omitted from further analysis. Null alleles were only indicated for Sco212 in the LO morph and the locus was retained in the dataset to maintain the statistical power.

None of the tests for deviation from HWE were significant before sequential Bonferroni corrections. Six pair-wise combinations of loci were significant for LD before sequential Bonferroni corrections, but only three remained significant after. Manual inspection revealed that the loci Sco218 and Sco204 displayed significant LD in all three morphs and the locus Sco218 were therefore omitted from the analyses.

Hence, the classification of the charr morphs by genetic assignment are based on the following seven microsatellite loci: SalF56SFU, SalP61SFU, SalJ81SFU, Sco204, Sco212, SMM17, and SMM22.

Table S4: Details of the nine microsatellite loci used to design the two PCR multiplexes for *S. alpinus.* The locus ID, PCR multiplex assignment (Mplx), concentration (Conc), fluorophor (Fph), alignment temperature (Ta), repeat motif (RM), and the source are given. Forward and reverse primers were used in equal concentrations.

| **Locus ID** | **Mplx** | **Conc (µM)** | **Fph** | **Ta (°C)** | **RM** | **Source** |
| --- | --- | --- | --- | --- | --- | --- |
| SalF56SFU | 1 | 1.0 | NED | 60 | TG | (McGowan et al. 2004) |
| SalP61SFU | 1 | 1.5 | PET | 60 | CA | (McGowan et al. 2004) |
| SalJ81SFU | 2 | 1.5 | NED | 55 | GT | (McGowan et al. 2004) |
| Sco204 | 2 | 1.5 | 6-FAM | 55 | TCTA | (Dehaan and Ardren 2005) |
| Sco212 | 1 | 1.0 | PET | 60 | ATCT | (Dehaan and Ardren 2005) |
| Sco215 | 2 | 0.5 | NED | 55 | GA | (Dehaan and Ardren 2005) |
| Sco218 | 1 | 1.0 | VIC | 60 | GATA | (Dehaan and Ardren 2005) |
| SMM17 | 2 | 0.4 | VIC | 55 | CA | (Crane et al. 2004) |
| SMM22 | 2 | 0.5 | PET | 55 | TAGA | (Crane et al. 2004) |

**References Appendix S2**

Crane PA, Lewis CJ, Kretschmer EJ, Miller SJ, Spearman WJ, DeCicco AL, Lisac MJ, Wenburg JK (2004) Characterization and inheritance of seven microsatellite loci from Dolly Varden, *Salvelinus malma*, and cross-species amplification in Arctic char, *S. alpinus*. *Conserv.- Gen.-* 5, 737-741.

Dehaan PW, Ardren WR (2005) Characterization of 20 highly variable tetranucleotide microsatellite loci for bull trout (*Salvelinus confluentus*) and cross-amplification in other *Salvelinus* species. *Mol. Ecol. Notes* **5**, 582-585.

Guo SW, Thompson EA (1992) Performing the exact test of Hardy-Weinberg proportion for multiple alleles. *Biometrics* 48, 361-372.

McGowan CR, Davidson EA, Woram RA*, et al.* (2004) Ten polymorphic microsatellite markers from Arctic charr (*Salvelinus alpinus*): linkage analysis and amplification in other salmonids. *Anim. Gen.* 35, 479-481.

Rice WR (1989) Analyzing tables of statistical tests. *Evolution* 43, 223-225.

Rousset F (2007) Genepop'007: a complete reimplementation of the Genepop software for Windows and Linux. *Mol. Ecol. Res.* 8, 103-106.

Van Oosterhout C, Hutchinson WF, Wills DPM, Shipley P (2004) MICRO-CHECKER: software for identifying and correcting genotyping errors in microsatellite data. *Mol. Ecol. Notes* 4, 535-538.

**Appendix S3 (genetics).**

**Results from the classification of charr morphs using genetic assignment**

Table S5: Summary statistics from the STRUCTURE analysis of adult Arctic charr morphs. The bold values indicate the most likely number of *K* clusters (*K* = 3) in the dataset.

| *K* | Reps | Mean LnP(*K*) | Stdev LnP(*K*) | *ΔK* |
| --- | --- | --- | --- | --- |
| 1 | 10 | -3914.3 | 0.7 | - |
| 2 | 10 | -3463.9 | 17.0 | 5.6 |
| **3** | **10** | **-3109.2** | **0.7** | **614.1** |
| 4 | 10 | -3155.9 | 21.6 | 0.7 |
| 5 | 10 | -3188.5 | 21.3 | 3.6 |
| 6 | 10 | -3297.9 | 60.0 | - |

**
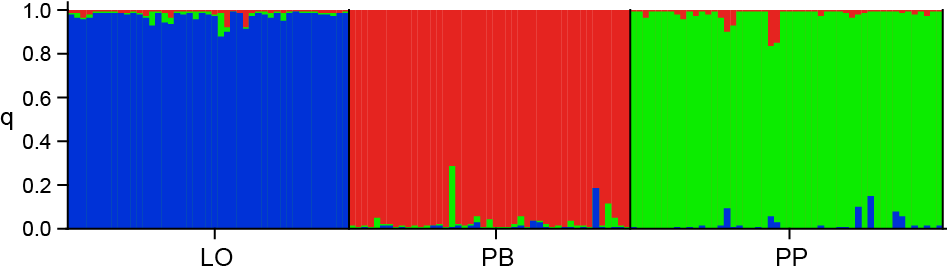
**

Fig. S3: Genetic structure of the three phenotypic (adult) charr morphs from Lake Skogfjordvatn as inferred by STRUCTURE. Black lines separate individuals from different phenotypic morphs (labelled below) and each individual is represented by a thin horizontal line which is partitioned into *K-*coloured segments representing individual’s estimated membership fractions in *K* clusters (*K* = 3). For each cluster the mean values of ln Pr(*X* |*K*) and Δ*K* for each value of *K* are found in Table S5.

**
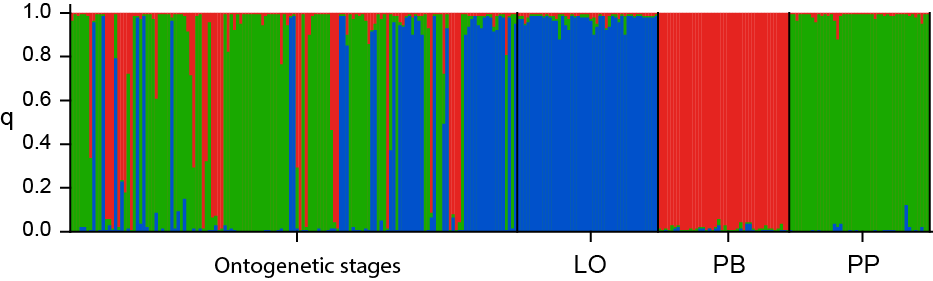
**

Fig. S4:

Genetic structure of all the charr individuals from Lake Skogsfjordvatn as inferred by STRUCTURE. Black lines separate individuals from different phenotypic morphs (labelled below) and each individual is represented by a thin horizontal line. which is partitioned into *K-*coloured segments representing individual’s estimated membership fractions in *K* clusters (*K* = 3). For each cluster. the mean values of ln Pr(*X* |*K*) and Δ*K* for each value of *K* are found in Table S5.

Table S6: Results from the assignment of the ontogenetic stages to the three adult reference populations (LO-, PB-, PP-morphs) of Arctic charr, using three different assignment approaches, rank and probability testing (GENECLASS2) and STRUCTURE. The consensus is given for situations where at least two out of the three genetic approaches assigned an individual (Genetic) to one of the reference populations. The consensus from the phenotypic classification (phenotype), are also given. Bold values indicate situations where one of the three assignment approaches did not correspond to the other two approaches. Values in italics indicate situations where the phenotypic and genotypic consensus of morph type did not correspond.

| **ASSIGNED SAMPLE** | **RANK TEST**  **(Rank, scores)** | | **PROBABILITY TESTING (probabilities)** | | | **STRUCTURE (q-values)** | | | **CONSENSUS** | |  |
| --- | --- | --- | --- | --- | --- | --- | --- | --- | --- | --- | --- |
|  | Rank | Score | LO | PB | PP | LO | PB | PP | Genetic | Phenotype | |
| Skg11021 | LO | 100 | 0.509 | 0.000 | 0.000 | 0.958 | 0.035 | 0.007 | LO | LO | |
| Skg11379 | LO | 100 | 0.947 | 0.000 | 0.000 | 0.983 | 0.011 | 0.006 | *LO* | *PB* | |
| Skg11383 | LO | 100 | 0.774 | 0.000 | 0.000 | 0.989 | 0.005 | 0.007 | LO | LO | |
| Skg11764 | LO | 100 | 0.979 | 0.000 | 0.000 | 0.988 | 0.005 | 0.007 | LO | LO | |
| Skg11765 | LO | 100 | 0.649 | 0.000 | 0.000 | 0.987 | 0.007 | 0.006 | LO | LO | |
| Skg11766 | LO | 100 | 0.419 | 0.004 | 0.000 | 0.852 | 0.098 | 0.049 | LO | LO | |
| Skg12014 | LO | 100 | 0.202 | 0.000 | 0.000 | 0.918 | 0.077 | 0.005 | LO | LO | |
| Skg12015 | LO | 100 | 0.340 | 0.000 | 0.000 | 0.928 | 0.049 | 0.024 | LO | LO | |
| Skg12086 | LO | 100 | 0.445 | 0.001 | 0.000 | 0.963 | 0.012 | 0.026 | LO | LO | |
| Skg12114 | LO | 100 | 0.030 | 0.000 | 0.000 | 0.946 | 0.042 | 0.012 | LO | LO | |
| Skg12115 | LO | 100 | 0.110 | 0.000 | 0.000 | 0.937 | 0.057 | 0.006 | LO | LO | |
| Skg12116 | LO | 100 | 0.264 | 0.000 | 0.000 | 0.986 | 0.010 | 0.005 | LO | LO | |
| Skg12117 | LO | 100 | 0.254 | 0.000 | 0.000 | 0.990 | 0.004 | 0.006 | LO | LO | |
| Skg12118 | LO | 100 | 0.324 | 0.000 | 0.000 | 0.900 | 0.016 | 0.084 | LO | LO | |
| Skg12119 | LO | 100 | 0.892 | 0.000 | 0.000 | 0.988 | 0.006 | 0.006 | LO | LO | |
| Skg12120 | LO | 100 | 0.534 | 0.000 | 0.000 | 0.969 | 0.012 | 0.019 | LO | LO | |
| Skg12125 | LO | 100 | 0.610 | 0.001 | 0.001 | 0.900 | 0.008 | 0.092 | LO | LO | |
| Skg12154 | LO | 100 | 0.671 | 0.000 | 0.000 | 0.992 | 0.004 | 0.004 | LO | LO | |
| Skg12700 | LO | 100 | 0.051 | 0.000 | 0.000 | 0.905 | 0.006 | 0.089 | LO | LO | |
| Skg12714 | LO | 100 | 0.090 | 0.000 | 0.000 | 0.989 | 0.006 | 0.005 | LO | LO | |
| Skg12715 | LO | 100 | 0.898 | 0.000 | 0.000 | 0.980 | 0.006 | 0.015 | LO | LO | |
| Skg12719 | LO | 100 | 0.624 | 0.000 | 0.000 | 0.924 | 0.008 | 0.068 | LO | LO | |
| Skg12720 | LO | 100 | 0.442 | 0.000 | 0.000 | 0.990 | 0.004 | 0.006 | LO | LO | |
| Skg12721 | LO | 100 | 0.985 | 0.000 | 0.000 | 0.981 | 0.005 | 0.014 | LO | LO | |
| Skg12727 | LO | 100 | 0.424 | 0.000 | 0.000 | 0.951 | 0.007 | 0.042 | LO | LO | |
| Skg11242 | PB | 100 | 0.001 | 0.056 | 0.000 | 0.010 | 0.939 | 0.052 | PB | PB | |
| Skg11243 | PB | 100 | 0.004 | 0.353 | 0.000 | 0.031 | 0.941 | 0.028 | PB | PB | |
| Skg11244 | PB | 100 | 0.001 | 0.960 | 0.000 | 0.008 | 0.981 | 0.011 | PB | PB | |
| Skg11247 | PB | 100 | 0.004 | 0.073 | 0.001 | 0.013 | 0.974 | 0.013 | PB | PB | |
| Skg11387 | PB | 61 | 0.107 | 0.432 | 0.043 | 0.089 | **0.387** | **0.525** | *PB* | *PP* | |
| Skg11598 | PB | 98 | 0.001 | 0.042 | 0.001 | 0.021 | 0.706 | 0.273 | PB | PB | |
| Skg11602 | PB | 100 | 0.000 | 0.201 | 0.000 | 0.006 | 0.981 | 0.013 | PB | PB | |
| Skg11603 | PB | 99 | 0.000 | 0.077 | 0.001 | 0.007 | 0.674 | 0.320 | PB | PB | |
| Skg11607 | PB | 100 | 0.017 | 0.726 | 0.004 | 0.014 | 0.930 | 0.056 | PB | PB | |
| Skg11608 | PB | 100 | 0.001 | 0.116 | 0.000 | 0.031 | 0.925 | 0.044 | PB | PB | |
| Skg11609 | PB | 100 | 0.000 | 0.906 | 0.000 | 0.006 | 0.988 | 0.006 | PB | PB | |
| Skg11610 | PB | 100 | 0.001 | 0.540 | 0.000 | 0.007 | 0.982 | 0.011 | PB | PB | |
| Skg11674 | PB | 91 | 0.022 | 0.570 | 0.028 | 0.015 | 0.705 | 0.279 | PB | PB | |
| Skg11675 | PB | 100 | 0.000 | 0.862 | 0.000 | 0.005 | 0.989 | 0.007 | PB | PB | |
| Skg11679 | PB | 100 | 0.000 | 0.009 | 0.000 | 0.012 | 0.976 | 0.012 | PB | PB | |
| Skg11691 | PB | 100 | 0.000 | 0.156 | 0.001 | 0.018 | **0.534** | **0.448** | PB | PB | |
| Skg11692 | PB | 100 | 0.000 | 0.212 | 0.000 | 0.015 | 0.953 | 0.032 | PB | PB | |
| Skg11694 | PB | 100 | 0.002 | 0.988 | 0.000 | 0.005 | 0.985 | 0.009 | PB | PB | |
| Skg12033 | PB | 100 | 0.000 | 0.295 | 0.000 | 0.009 | 0.984 | 0.007 | PB | PB | |
| Skg12151 | PB | 100 | 0.010 | 0.478 | 0.001 | 0.070 | 0.914 | 0.017 | PB | PB | |
| Skg12695 | PB | 100 | 0.000 | 0.737 | 0.000 | 0.006 | 0.989 | 0.005 | PB | PB | |
| Skg12696 | PB | 100 | 0.008 | 0.056 | 0.000 | 0.067 | 0.917 | 0.016 | PB | PB | |
| Skg12697 | PB | 100 | 0.000 | 0.480 | 0.000 | 0.006 | 0.990 | 0.004 | PB | PB | |
| Skg12698 | PB | 100 | 0.006 | 0.707 | 0.001 | 0.011 | 0.953 | 0.036 | PB | PB | |
| Skg11380 | PP | 100 | 0.014 | 0.037 | 0.234 | 0.041 | 0.022 | 0.937 | PP | PP | |
| Skg11384 | PP | 100 | 0.005 | 0.003 | 0.423 | 0.026 | 0.007 | 0.966 | PP | PP | |
| Skg11385 | PP | 100 | 0.007 | 0.097 | 0.557 | 0.004 | 0.006 | 0.990 | PP | PP | |
| Skg11386 | PP | 100 | 0.001 | 0.155 | 0.637 | 0.005 | 0.022 | 0.973 | PP | PP | |
| Skg11390 | PP | 100 | 0.038 | 0.001 | 0.399 | 0.005 | 0.005 | 0.991 | PP | PP | |
| Skg11391 | PP | 100 | 0.017 | 0.000 | 0.291 | 0.015 | 0.004 | 0.980 | PP | PP | |
| Skg11397 | PP | 100 | 0.000 | 0.002 | 0.274 | 0.005 | 0.005 | 0.990 | PP | PP | |
| Skg11399 | PP | 100 | 0.006 | 0.008 | 0.590 | 0.004 | 0.005 | 0.991 | PP | PP | |
| Skg11452 | PP | 100 | 0.000 | 0.000 | 0.169 | 0.014 | 0.004 | 0.981 | PP | PP | |
| Skg11472 | PP | 100 | 0.009 | 0.000 | 0.028 | 0.100 | 0.007 | 0.893 | PP | PP | |
| Skg11541 | PP | 100 | 0.002 | 0.001 | 0.600 | 0.004 | 0.004 | 0.992 | PP | PP | |
| Skg11542 | PP | 100 | 0.014 | 0.002 | 0.175 | 0.157 | 0.008 | 0.835 | PP | PP | |
| Skg11596 | PP | 100 | 0.003 | 0.016 | 0.320 | 0.006 | 0.085 | 0.910 | PP | PP | |
| Skg11597 | PP | 99 | 0.000 | **0.216** | 0.083 | 0.007 | 0.282 | 0.711 | PP | PP | |
| Skg11599 | PP | 100 | 0.001 | 0.147 | 0.603 | 0.004 | 0.009 | 0.988 | PP | PP | |
| Skg11600 | PP | 100 | 0.004 | 0.004 | 0.565 | 0.020 | 0.005 | 0.975 | PP | PP | |
| Skg11604 | PP | 100 | 0.307 | 0.322 | 0.487 | 0.012 | 0.042 | 0.946 | PP | PP | |
| Skg11611 | PP | 100 | 0.012 | 0.001 | 0.444 | 0.029 | 0.005 | 0.966 | PP | PP | |
| Skg11612 | PP | 100 | 0.004 | **0.352** | 0.136 | 0.008 | 0.174 | 0.817 | PP | PP | |
| Skg11613 | PP | 100 | 0.030 | 0.004 | 0.148 | 0.016 | 0.006 | 0.978 | PP | PP | |
| Skg11614 | PP | 100 | 0.001 | 0.023 | 0.173 | 0.008 | 0.074 | 0.918 | PP | PP | |
| Skg11615 | PP | 100 | 0.011 | 0.004 | 0.976 | 0.004 | 0.005 | 0.991 | PP | PP | |
| Skg11616 | PP | 100 | 0.007 | 0.070 | 0.474 | 0.006 | 0.045 | 0.950 | PP | PP | |
| Skg11617 | PP | 100 | 0.004 | 0.005 | 0.865 | 0.005 | 0.004 | 0.991 | PP | PP | |
| Skg11618 | PP | 100 | 0.026 | 0.012 | 0.837 | 0.005 | 0.005 | 0.991 | PP | PP | |
| Skg11619 | PP | 100 | 0.018 | 0.009 | 0.596 | 0.006 | 0.005 | 0.989 | PP | PP | |
| Skg11620 | PP | 100 | 0.006 | 0.010 | 0.845 | 0.005 | 0.005 | 0.990 | PP | PP | |
| Skg11621 | PP | 100 | 0.000 | 0.013 | 0.248 | 0.005 | 0.007 | 0.989 | PP | PP | |
| Skg11622 | PP | 100 | 0.047 | 0.003 | 0.571 | 0.007 | 0.005 | 0.988 | PP | PP | |
| Skg11624 | PP | 100 | 0.014 | 0.034 | 0.353 | 0.005 | 0.052 | 0.943 | PP | PP | |
| Skg11625 | PP | 100 | 0.019 | 0.008 | 0.359 | 0.010 | 0.008 | 0.982 | PP | PP | |
| Skg11626 | PP | 100 | 0.015 | 0.010 | 0.698 | 0.009 | 0.006 | 0.986 | PP | PP | |
| Skg11627 | PP | 100 | 0.006 | 0.001 | 0.516 | 0.005 | 0.004 | 0.991 | PP | PP | |
| Skg11630 | PP | 100 | 0.002 | 0.003 | 0.230 | 0.005 | 0.005 | 0.991 | PP | PP | |
| Skg11631 | PP | 100 | 0.061 | 0.000 | 0.165 | 0.015 | 0.006 | 0.980 | PP | PP | |
| Skg11632 | PP | 100 | 0.000 | 0.001 | 0.009 | 0.016 | 0.232 | 0.753 | PP | PP | |
| Skg11633 | PP | 100 | 0.003 | 0.001 | 0.781 | 0.004 | 0.004 | 0.991 | PP | PP | |
| Skg11634 | PP | 100 | 0.000 | 0.007 | 0.194 | 0.004 | 0.054 | 0.941 | PP | PP | |
| Skg11678 | PP | 100 | 0.034 | 0.004 | 0.591 | 0.009 | 0.005 | 0.985 | PP | PP | |
| Skg11680 | PP | 100 | 0.009 | 0.044 | 0.169 | 0.006 | 0.093 | 0.901 | PP | PP | |
| Skg11681 | PP | 100 | 0.000 | 0.017 | 0.275 | 0.005 | 0.008 | 0.987 | PP | PP | |
| Skg11682 | PP | 100 | 0.002 | 0.002 | 0.410 | 0.007 | 0.005 | 0.988 | PP | PP | |
| Skg11683 | PP | 100 | 0.023 | 0.005 | 0.180 | 0.019 | 0.007 | 0.974 | PP | PP | |
| Skg11684 | PP | 100 | 0.006 | 0.087 | 0.548 | 0.005 | 0.006 | 0.989 | PP | PP | |
| Skg11685 | PP | 100 | 0.009 | 0.017 | 0.435 | 0.005 | 0.007 | 0.987 | PP | PP | |
| Skg11686 | PP | 100 | 0.006 | 0.001 | 0.143 | 0.008 | 0.005 | 0.987 | PP | PP | |
| Skg11775 | PP | 100 | 0.011 | 0.006 | 0.878 | 0.004 | 0.004 | 0.991 | PP | PP | |
| Skg11776 | PP | 100 | 0.090 | 0.005 | 0.121 | 0.023 | 0.028 | 0.949 | PP | PP | |
| Skg11777 | PP | 100 | 0.080 | 0.018 | 0.524 | 0.010 | 0.006 | 0.984 | PP | PP | |
| Skg11778 | PP | 100 | 0.071 | 0.052 | 0.509 | 0.010 | 0.008 | 0.982 | PP | PP | |
| Skg11779 | PP | 100 | 0.000 | 0.002 | 0.216 | 0.004 | 0.005 | 0.991 | PP | PP | |
| Skg11780 | PP | 100 | 0.046 | 0.062 | 0.142 | 0.017 | 0.031 | 0.952 | PP | PP | |
| Skg11781 | PP | 100 | 0.001 | 0.007 | 0.108 | 0.010 | 0.142 | 0.847 | PP | PP | |
| Skg12021 | PP | 100 | 0.090 | 0.015 | 0.740 | 0.005 | 0.005 | 0.990 | PP | PP | |
| Skg12022 | PP | 100 | 0.089 | 0.007 | 0.152 | 0.054 | 0.024 | 0.921 | PP | PP | |
| Skg12031 | PP | 100 | 0.002 | 0.013 | 0.982 | 0.004 | 0.004 | 0.992 | PP | PP | |
| Skg12082 | PP | 86 | **0.259** | 0.000 | 0.026 | 0.374 | 0.007 | 0.719 | PP | PP | |
| Skg12091 | PP | 100 | 0.002 | 0.016 | 0.259 | 0.010 | 0.008 | 0.982 | PP | PP | |
| Skg12143 | PP | 100 | 0.005 | 0.017 | 0.566 | 0.007 | 0.005 | 0.988 | PP | PP | |
| Skg12148 | PP | 100 | 0.000 | 0.005 | 0.063 | 0.009 | 0.069 | 0.921 | PP | PP | |
| Skg12158 | PP | 100 | 0.028 | 0.007 | 0.238 | 0.006 | 0.006 | 0.987 | PP | PP | |
| Skg12162 | PP | 100 | 0.001 | 0.003 | 0.212 | 0.005 | 0.006 | 0.989 | PP | PP | |
| Skg12699 | PP | 100 | 0.009 | 0.003 | 0.601 | 0.010 | 0.005 | 0.985 | PP | PP | |
| Skg12722 | PP | 100 | 0.000 | 0.032 | 0.042 | 0.011 | 0.186 | 0.802 | PP | PP | |

Table S7: Pair-wise entries of FST among the three Arctic charr morphs as inferred by Arlequin 3.5.3 (Excoffier and Lischer, 2010). All estimates returned highly significant (P < 0.001) using 10,000 permutations.

Distance method: No. of different alleles (FST)

| **LO-PB** | 0.157 |
| --- | --- |
| **LO-PP** | 0.236 |
| **PB-PP** | 0.268 |

Reference

Excoffier L, Lischer HEL (2010) Arlequin suite ver 3.5: A new series of programs to perform population genetics analyses under Linux and Windows. Mol. Ecol. Res. 10: 564-567.
